# Statistical modeling of days alive out-of-hospital: An illustration using the SSU trial

**DOI:** 10.1017/cts.2026.10786

**Published:** 2026-07-07

**Authors:** Xin Wu, David A. Berger, Jason J. Bischof, Douglas Char, Deborah Diercks, Erik P. Hess, Alan E. Jones, Kathleen A. Lane, Phillip Levy, Xiaochun Li, Joseph B. Miller, Simon A. Mahler, Susan J. Pressler, Arvind Venkat, Peter S. Pang, Sean Collins, Bryan S. Blette

**Affiliations:** 1 Department of Biostatistics, Vanderbilt University Medical Center, Nashville, TN, USA; 2 Department of Emergency Medicine, William Beaumont Hospital, Royal Oak, MI, USA; 3 Department of Emergency Medicine, The Ohio State University College of Medicine, Columbus, OH, USA; 4 Department of Emergency Medicine, Washington University in St Louis, St Louis, MO, USA; 5 Department of Emergency Medicine, The University of Texas Southwestern Medical Center, Dallas, TX, USA; 6 Department of Emergency Medicine, Vanderbilt University Medical Center, Nashville, TN, USA; 7 Department of Emergency Medicine, The University of Mississippi Medical Center, Jackson, MS, USA; 8 Department of Biostatistics and Health Data Science, Indiana University School of Medicine,, Indianapolis, IN, USA; 9 Department of Emergency Medicine and Integrative Biosciences Center, Wayne State University, Detroit, MI, USA; 10 Department of Emergency Medicine, Henry Ford Health System, Detroit, MI, USA; 11 Department of Emergency Medicine, Wake Forest University School of Medicine, Winston Salem, NC, USA; 12 Indiana University School of Nursing, Indianapolis, IN, USA; 13 Department of Emergency Medicine, Allegheny Health Network, Pittsburgh, PA, USA; 14 Indiana University School of Medicine, Indianapolis, IN, USA

**Keywords:** cardiovascular health, days alive out of Hospital, G-computation, ordinal regression, skewed data

## Abstract

**Background:**

Days alive out-of-hospital (DAOOH) is a well-accepted outcome in clinical research, as it summarizes both hospitalization and mortality. However, its distribution is often skewed, making accurate modeling challenging in practice. We utilized data from a randomized trial to discuss estimands and statistical methods of interest for DAOOH.

**Methods:**

We examined several estimands and statistical models targeting these estimands, including linear regression, gamma regression, ordinal regression, and the probabilistic index model. Comparison was based on interpretability, precision, and model fit. We adopted G-computation to assess causal effects leveraging different regression models to target a simple unified estimand.

**Results:**

For unadjusted analyses, the Mann–Whitney *U*-test is a simple and assumption-light approach, which can be paired with a probabilistic index to enhance interpretation. For adjusted analyses, while the mean difference and mean ratio are intuitive, the methods targeting them (linear and gamma regression) showed sub-optimal model fit. Conversely, ordinal regression showed a superior fit, but its odds ratio estimand is difficult to interpret. Pairing gamma or ordinal regression with g-computation facilitates estimation of a marginal causal mean difference estimand under certain assumptions, allowing for both a good model fit and interpretability.

**Conclusions:**

Our findings illustrate trade-offs of estimands and model choices for DAOOH, providing guidance for method selection. Based on data from this randomized trial, the Mann–Whitney *U*-test is recommended for unadjusted analyses. Ordinal regression paired with g-computation provides the best balance of precision, model fit, and interpretability to report an adjusted effect.

## Introduction

Hospital-free days have become increasingly popular in acute care trials because they account for both morbidity and mortality [1–3]. One version of free days, days alive out-of-hospital (DAOOH) is an acceptable outcome for low-risk acute heart failure trials. DAOOH is defined as the number of days a patient is not hospitalized and alive within a specified period after randomization. Previous studies have discussed the clinical validity of DAOOH in association with hospitalization burden [4, 5] and its potential as a composite outcome to enhance statistical power to detect treatment efficacy [5].

Importantly, DAOOH integrates the frequency and duration of hospitalization (including rehospitalization), as well as mortality, into a single outcome [6]. It is a distinct outcome from conventional time-to-event endpoints like time until discharge. For patients who die during a trial, DAOOH is often set to 0 to indicate the worst health status (i.e., a worse outcome than someone hospitalized for the full period but still alive at time of assessment). Even when death occurs later during the follow-up period, DAOOH can differentiate these patients from those who experienced hospital revisits at the beginning but survived and remained out of hospital [5]. For patients with multiple hospitalizations, DAOOH captures not only whether rehospitalization occurred, but also how often and for how long. Thus, it differentiates patients with repeated hospital stays from those with a single visit. Patients experiencing fewer hospital revisits and better health status will have higher DAOOH. This further distinguishes DAOOH from a traditional time-to-event formulation, where recurrent hospitalizations might be missed or inadequately summarized. Hospital stays are associated with severe symptoms, in-hospital adverse events and lower quality of life [7, 8]. Patients prefer shorter hospital stays as long as an earlier discharge does not increase risk of death [7]. Therefore, DAOOH is closely related to the patient’s quality of life and is a patient-centered outcome.

**Figure 1.**
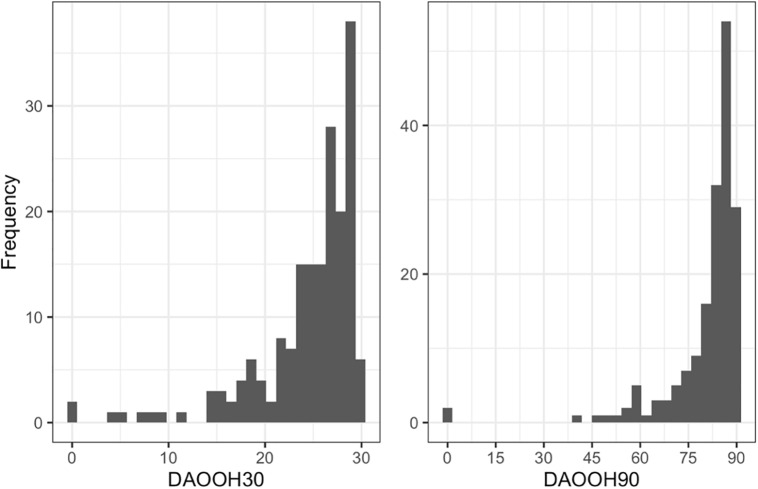
Distribution of the outcome variables in the SSU trial: DAOOHR30 (left) and DAOOHR90 (right). DAOOH30, days alive out of hospital within 30 days after hospital discharge; DAOOH90, days alive out of hospital within 90 days after hospital discharge.

For a period of 30 days after discharge, approximately 20–25% of acute heart failure patients are rehospitalized [6, 9]. DAOOH is thus often left-skewed in this population as a large proportion of patients survive with no or few hospital revisits after discharge. In addition, initial hospitalizations often only last a few days in the low-risk population. Consequently, the DAOOH values tend to concentrate near 27–29 days within a 30-day period. In the recently completed short-stay units (SSU) trial comparing SSU vs routine hospital admission for low-risk acute heart failure patients [6], DAOOH after randomization clustered around 30 and 90 days within the 30-day period and 90-day period respectively (Figure 1). Furthermore, DAOOH is subject to ceiling and floor values because of potential deaths and limited follow-up duration.

Each of these outcome characteristics make DAOOH challenging to model. This study aims to explore different statistical tests and models for DAOOH, including additive models such as linear regression and multiplicative models such as gamma regression. We further illustrate and compare the models using the SSU trial as an example of data from a completed clinical trial. Because 30 days after discharge is an important window for re-hospitalization of patients with an acute medical problem [7], we center the discussion on DAOOH within a 30-day period. These results will provide guidance in writing and executing statistical analysis plans for future trials for investigators using DAOOH as a primary outcome.

## Material and methods

### Target estimands

The ICH E9 guidelines for clinical trials [10, 11] recommend clearly specifying the estimand of interest (i.e., a formal definition of the treatment effect to be estimated) and then choosing an estimator which is valid for that estimand. Estimands have several components, including describing the population, treatment conditions, endpoint, how intercurrent events will be handled, and the summary measure [12]. The focus of this manuscript is on the final component and the two arm study setting. There is no standard estimand recommended for trials that use DAOOH as a primary outcome, but there are several potential estimands of interest, each with strengths and limitations regarding interpretability and ease of estimation.

A common estimand of interest in trials is the mean difference in the primary outcome across arms, sometimes referred to as the average treatment effect on the difference scale. An advantage of this estimand is that it is easy to interpret and familiar to investigators and patients. A weakness is that it can be sensitive to distributions with outliers, and that standard methods naturally targeting the mean difference may not perform well with heavily skewed data. A patient would interpret the estimand as the average number of additional days that they would be able to safely be home after receiving the intervention (accounting for death as previously described).

A second potential estimand is the mean ratio or fold change across arms, i.e., the ratio of average DAOOH between the treatment group and the control group. Advantages include its ability to accommodate skewed outcomes well (particularly right-skewed data) and its utility when the intervention is expected to have a proportional effect (e.g., reduction of more hospital days for individuals who would have longer hospital stays when assigned to control). A weakness is that while some investigators are familiar with mean ratios, this estimand may be less interpretable to both investigators and patients. Because this quantity is easier to estimate with right-skewed data than left-skewed data, we consider this estimand for a transformed (30 – DAOOH) outcome named death or hospital days (DOHD), which is equivalent to a composite outcome of days *in* hospital with death equal to 30.

Another potential estimand is the odds ratio: the ratio of cumulative odds of DAOOH being in a “higher category” under intervention than under control. This estimand treats the outcome as ordinal and is not directly interpretable on the day scale for an outcome like DAOOH. However, continuous outcomes can be considered ordinal if there exists a clear hierarchical relationship between the different values of the outcome (e.g., higher values indicates better (or worse) outcomes), and recent work has highlighted that cumulative probability models which target odds ratios can be a surprisingly robust modeling choice for skewed continuous data [13]. It is a meaningful choice when the relative positions of DAOOH is more of interest or when the optimal transformation for modeling DAOOH as a continuous variable is unclear [14]. Importantly, the odds ratio is a relative ordering measure and should not be interpreted as a direct difference in days. For this reason, it may be difficult for both investigators and patients to interpret in practice and benefits from reporting marginal effects on the original scale to enhance interpretation when possible.

The last estimand we consider is the Mann–Whitney treatment effect: the probability that DAOOH of a random observation from the treatment group is larger than that from a random observation of the control group [15]. It is useful for comparing the overall distribution of DAOOH between two groups. An advantage is that it can be targeted with methods which make no or few parametric assumptions, which may be ideal for outcomes with unusual distributions like DAOOH. However, interpretation can be challenging because it is purely built on probability and cannot be transformed back to the original scale to quantify how many days the intervention extends DAOOH. Further, it may understate the impact of interventions which have a large effect on a small subset of patients.

These estimands are not exhaustive but represent a set of target quantities which are estimable and used in practice for outcomes like DAOOH. There are other possible ways to define a quantity of interest, including the median and quantile treatment effects, which capture differences in specific quantiles of the outcome distribution rather than in the mean. These are used infrequently for DAOOH and beyond the scope of this study; we refer to previous work describing such estimands [16, 17]. In the next section, we describe estimators and models which target each of the estimands considered.

### Modeling approaches

Commonly used tests and models can be applied to estimate the treatment effects defined by the estimands mentioned in the previous section. Some of these naturally target certain estimands, while others require proper transformation. We describe the models and their interpretation for each estimand. We also consider g-computation [18, 19], a technique which enables generation of a standardized treatment effect estimate on the desired scale of DAOOH regardless of the specified outcome model, under certain assumptions. The results of this subsection and the Results section will be based on the DAOOH after randomization within 30 days outcome reported in the SSU trial.

#### Mean difference

The *t*-test can be used to detect if there is a statistically significant difference between the means of two independent groups. In addition to testing a null hypothesis of no difference in mean DAOOH between arms, it corresponds to and is typically paired with the average treatment effect on the difference scale and a confidence interval. Linear regression also estimates the mean difference. The estimated treatment coefficient represents the treatment effect of SSU treatment, and can be interpreted as the difference between the average DAOOH in the two groups.

#### Mean ratio

Gamma regression enables the estimation of mean ratio in a response variable. To ensure model feasibility, we further transform the response variable into DOHD, defined as *DOHD* = 30 − *DAOOH*, where 30 represents the follow-up period length. Therefore, the gamma regression is in fact modeling how the SSU treatment affects the length of hospital stay with death set to the worst value of 30. Exponentiating the estimated treatment coefficient yields the treatment effect of SSU, interpreted as the mean ratio between the average DOHD (30 − DAOOH) of the two study arms.

#### Odds ratio

Ordinal regression is an extension of binomial regression to ordered categorical outcomes [20]. By using this model, the DAOOH is treated as an ordinal variable with each of its values as a category. Instead of investigating how the SSU treatment affects the average value of DAOOH, the model focuses on the influence of SSU treatment on relative ordering of DAOOH. The exponentiated coefficient represents the treatment effect of SSU on DAOOH and can be interpreted as the odds ratio of being in a higher DAOOH level (compared to a lower level) under intervention vs under control. While higher values are consistently considered better for DAOOH, making this interpretation meaningful, this framework can be poor for outcomes like blood pressure where the values that are considered better depend on where a patient is in the outcome distribution (although similar issues can exist when analyzing systolic blood pressure as a continuous outcome and considering a fixed effect size across the outcome distribution).

#### Mann–Whitney treatment effect

The Mann–Whitney *U*-test is a non-parametric test comparing the distribution of two independent samples by the difference in mean ranks of the two samples. The Mann–Whitney effect parameter, also called the probabilistic index, can be estimated in parallel to the *U*-test and represents the probability the DAOOH of a randomly selected subject in SSU group exceeds the DAOOH of that in the Hospitalization group [21, 22]. Inference of the parameter is achieved via a bootstrap procedure.

### Covariate incorporation

Whether baseline covariates are incorporated is a key consideration that affects both the choice of analytic method and the interpretation of the treatment effect estimate. In the SSU trial, several important pre-specified prognostic covariates were identified, including any emergency department visit or hospitalization in the past 6 months, systolic blood pressure, blood urea nitrogen value, troponin-I value, and b-type natriuretic peptide value measured at randomization. While covariate adjustment should improve precision in large samples, it can reduce precision in smaller trials if included covariates are only weakly predictive of the outcome.

In general, the *t*-test and Mann–Whitney *U* test do not directly adjust for covariates, and therefore primarily target unadjusted, marginal treatment comparisons. In contrast, regression-based approaches, including linear regression, gamma regression, ordinal regression, and the probabilistic index model (a model which targets a Mann–Whitney parameter), can incorporate baseline covariates. When covariates are included, these approaches yield conditional treatment effect estimates, so the estimated effect be interpreted as conditional on the covariates in the model. When no covariates are included, these regression-based methods may also be used to target marginal effects. Additionally, adjusting for pre-specified baseline covariates is recommended by the FDA to increase statistical power and precision, regardless of whether a marginal or conditional treatment effect is targeted [19].

### Targeting marginal estimands with g-computation

Lastly, we consider the method of g-computation, which allows for formal marginalization over adjustment covariates to target a marginal treatment effect. This method requires correct specification of the outcome regression model but is often robust to this assumption in the randomized trial setting [23]. This technique estimates the marginal causal effect by fitting an outcome model and making predictions for each observation under each treatment condition [18]. After obtaining the outcome predictions, they are transformed back to the desired comparison scale by applying specific formulas based on the transformation of interest. To illustrate, we combine the g-computation formula with each outcome regression described above (linear regression, gamma regression, ordinal regression) to target the marginal mean difference of DAOOH between two treatments as the estimand of interest. Under certain assumptions, this approach allows for using the best-fitting outcome model while still targeting an easily interpretable estimand, potentially alleviating some weaknesses of gamma and ordinal regression. This strategy was recently recommended in FDA guidelines as a robust approach to adjust for covariates [19].

### Illustration of methods using SSU trial

Each of the described methods are applied to data from the SSU trial with all analyses performed in R statistical software. The SSU trial has missing values in 2 of the 5 adjusted covariates, with missingness prevalence below 8%. We thus adopt multiple imputation [24–26] techniques to calculate the estimate for each model coefficient mentioned above assuming data are missing at random conditional on the other included covariates. The results are pooled using Rubin’s Rules [24]. This approach accounts for both within-imputation variance and between-imputation variance, thus reflecting the uncertainty introduced by missing data. For most models, imputation and pooling are carried out using the mice package [25]. Ordinal regression is fitted using the rms package in R [26], which fits cumulative probability models and can be used to analyze continuous outcomes treated as ordered categories. Although both packages accommodate multiple imputation within Rubin’s framework, their implementation differs slightly. G-computation is also implemented based on the pooled model fit. For the confidence interval of the g-computation result, we draw bootstrap samples from trial data and multiple imputation is used in each bootstrap sample to achieve a more valid result [27]. The multiple imputation is implemented using 15 imputed datasets in the mice package and a random seed of 123.

### Evaluation criteria

We compare all approaches in terms of interpretability. For the regression-based methods, we additionally assess precision and model fit. Interpretability is evaluated according to whether the corresponding test or model targets an estimand that is practical to interpret for clinicians and patients; while this is subjective, we consider whether results can be understood as differences in DAOOH on an additive scale to be a key marker of such interpretability. Precision is assessed using the width of the confidence intervals from the G-computation results, with narrower intervals indicating greater precision. For each regression model, we examine diagnostic plots to assess whether the model assumptions are reasonably satisfied to assess model fit. Specifically, we use residual-versus-fitted plots and quantile-quantile (Q–Q) plots. To facilitate comparison across regression models, we also calculate the root mean square error (RMSE) on the day scale. This provides a metric to measure how well the predicted values approximate the observed DAOOH outcomes. Table 1 summarizes the evaluation criteria.

**Table 1.** Evaluation criteria Table 1 long description.

Metric	Evaluation approach	Methods to be evaluated
Interpretability	Interpretation on difference scale for untransformed DAOOH	*T*-test, Mann–Whitney *U*-test, Linear Regression, Gamma Regression, Ordinal Regression, Probabilistic Index Model
Precision	Confidence interval width	Linear Regression, Gamma Regression, Ordinal Regression
Model fit	Diagnostic plots	Linear Regression, Gamma Regression, Ordinal Regression
	RMSE	

RMSE: Root mean squared error (at day scale).

## Results

Each of the considered tests and models are now illustrated using data from the SSU trial. Results are presented in Table 2. All regression coefficients were transformed to relevant estimands as described earlier. The linear regression model showed that average DAOOH after randomization in a 30-day period in the SSU group is estimated to be 1.35 days more than that in the Hospitalization group, conditional on the specified baseline covariates. The gamma regression model showed that the average DOHD (30 − DAOOH) after randomization in a 30-day period in the SSU group is 0.80 times that of the Hospitalization group, conditional on other covariates (i.e., a 20% shorter hospital stay on average). Both models produced estimates suggesting effectiveness of SSU treatment, but did not establish statistical significance at the α = 0.05 level.

**Table 2.** Model results Table 2 long description.

Method	Adjustment	Estimand	Effect type	Estimate (95% CI)	RMSE
***Additive model***					
*T*-test	Unadjusted	Mean difference	Marginal	1.44 (-0.16, 3.04)	
Linear regression	Adjusted	Mean difference	Conditional	1.35 (-0.17, 2.86)	5.11
***Multiplicative model***					
Gamma regression	Adjusted	Mean ratio	Conditional	0.80 (0.60, 1.06)	5.10
Ordinal regression	Adjusted	Odds ratio	Conditional	1.87 (1.12, 3.13)	5.12
***Probabilistic model***					
Mann–Whitney *U*-test	Unadjusted	Probabilistic index	Marginal	0.60 (0.51, 0.68)	
Probabilistic index model	Adjusted	Probabilistic index	Conditional	0.60 (0.51, 0.68)	

CI, confidence interval.

The ordinal regression model demonstrated the odds of DAOOH being in a higher category in the SSU group is 1.87 times that in the Hospitalization group, conditional on other covariates. The PIM model showed that the probability that a randomly selected subject in the SSU treatment group has a higher DAOOH than a randomly selected subject from the Hospitalization group is equal to 0.60, conditional on other covariates. These two models showed statistically significant results at the *α* = 0.05 level (note that the null hypothesis for the PIM model is that the probabilistic index is equal to 0.5), although interpretation of the confidence intervals shows similar uncertainty to linear or gamma regression.

**Figure 2.**
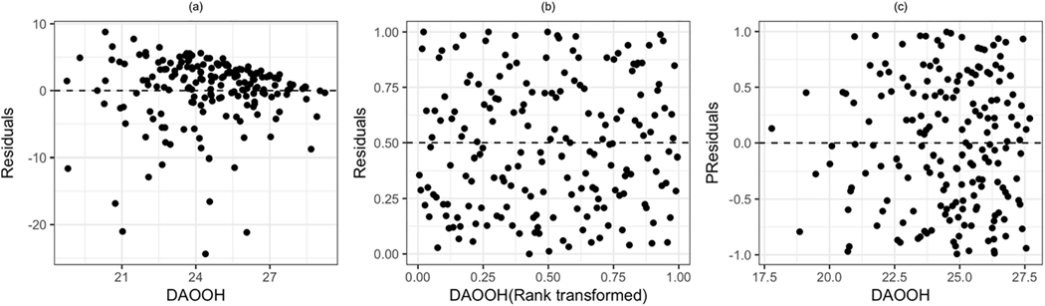
Figure 2 long description. Residual-by-fitted-value plots from different regression models. Centering around zero and homoskedasticity indicate better fits. (a) Residuals are from linear regression and the fitted value is DAOOH. (b) Simulated residuals are from gamma regression and the fitted DOHD (30 − DAOOH) is transformed to DAOOH. There exists a minor quantile deviation at the 0.25 quantile. (c) PResiduals are from ordinal regression and the fitted value is DAOOH. DAOOH, days alive out of hospital.

**Figure 3.**
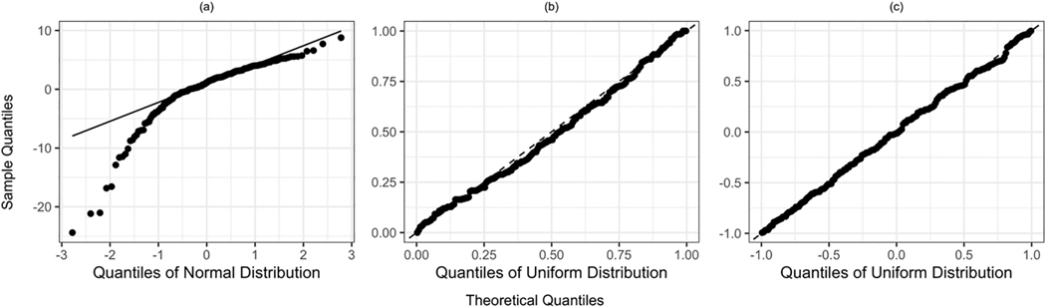
Figure 3 long description. Quantile-Quantile plots from different regression models. Alignment with the diagonal line indicates better fit. (a) Linear regression model. (b) Gamma regression model. (c) Ordinal regression models.

These models fitted the data differently and the quality of their fits should be evaluated. For convenience, diagnostic analysis was conducted based on one complete dataset produced by multiple imputation. A residual vs fitted value plot of linear regression showed spreading variance as the fitted value increases, indicating heteroscedasticity and that the linear model’s assumption of constant variance may be violated (Figure 2). Also, its Quantile-Quantile-plot (QQ plot) indicated that normality of residuals likely does not hold. The residuals of gamma regression were randomly distributed with the exception of a minor quantile deviation at the 0.25 quantile (Figure 2). The points of the QQ-plot for gamma regression mostly fell on a straight line, indicating that it fit the observed data distribution better than linear regression (Figure 3). The ordinal regression model produced a residual plot with no clear pattern, with points randomly spread around zero. Its performance was robust even at extreme values or in areas with sparse data. The QQ-plot of ordinal regression showed good agreement between the empirical and theoretical distributions, with points aligning along the diagonal (Figure 3). This suggests that the ordinal regression fits the observed data distribution better than the other two regression models and leaves less unexplained structure in the data. The ordinal regression relies on the proportional odds assumption, which implies that the treatment effect is approximately constant across outcome cutoffs. This assumption is assessed using the diagnostic tools provided in the rms package, and the results suggest no meaningful violation of the assumption. Details of the assessment are provided in Supplementary Figure S1.

The three regression models perform similarly in predicting the observed values. As shown in Table 2, gamma regression produced the lowest RMSE of 5.10, followed by ordinal regression (RMSE = 5.11) and linear regression (RMSE = 5.12). These negligible differences provide little evidence of meaningful difference in how well the three models predict the observed DAOOH.

The *t*-test and the Mann–Whitney *U*-test results are also displayed in Table 2. The *t*-test showed that average DAOOH after randomization in a 30-day period in the SSU group is estimated to be 1.44 days more than in the Hospitalization group and did not establish statistical significance at the 0.05 level. The Mann–Whitney *U*-test showed that the probability that a randomly selected subject in the SSU treatment group has a higher DAOOH than a randomly selected subject from the Hospitalization group is equal to 0.60. The Mann–Whitney *U*-test established statistical significance at the 0.05 level, in line with the findings of the SSU trial. The results obtained from the *t*-test were comparable to those of the linear regression model, and the results obtained from the Mann–Whitney *U*-test were comparable to those of the PIM model. Thus, the marginal and conditional treatment effects within each method pair were of similar magnitude.

G-computation results leveraging three different regression models yielded similar results to each other and to the original linear regression estimation results (Table 3). Recall that each method is now targeting the marginal mean difference in DAOOH across arms. For linear regression, g-computation reflects a standardization procedure that yields nearly identical results to the conditional estimate (and indeed for targeting the marginal mean difference, one can standardize without making predictions under each treatment condition to yield identical results in practice). All models estimated that the SSU treatment provides at least one additional DAOOH than Hospitalization. The ordinal regression produced the narrowest 95% confidence interval (width = 2.81), followed by linear regression (width = 3.11) and gamma regression (width = 3.31). At the same time, the ordinal regression produced the largest estimated effect size. Although the 95% CI from these regression models differed with respect to statistical significance, their lower confidence bounds were all close to 0. Additionally, with 184 observations, the sample size is relatively modest. Therefore, any difference in significance should be interpreted cautiously.

**Table 3.** G-computation results Table 3 long description.

Method	Estimand	Effect type	Estimate (95% CI)
***Additive model***			
Linear regression	Standardized mean difference	Marginal	1.37 (−0.07, 3.04)
***Multiplicative model***			
Gamma regression	Standardized mean difference	Marginal	1.23 (−0.38, 2.93)
Ordinal regression	Standardized mean difference	Marginal	1.43 (0.01, 2.82)

CI, confidence interval.

## Discussion

In this study, we aimed to find a balance between interpretable estimands and optimal statistical methods for analyzing and interpreting a trial treatment effect on DAOOH. We considered both parametric and non-parametric methods. Among all the considered estimands, the mean difference and mean ratio are likely easier to interpret because they are more familiar to investigators and patients alike. The odds ratio, though less interpretable, is one of the standard measures in clinical research. However, it can be difficult to interpret when used for continuous-valued outcomes. The probabilistic index is likely less familiar to both clinicians and patients. While the odds ratio and probabilistic index are both ultimately based on probability, the odds ratio expresses the relative odds and is more closely integrated in regression models and clinical guidelines.

For adjusted methods, models targeting the mean difference or mean ratio, such as linear regression and gamma regression, may be preferred in analysis plans. In addition, methods that allow treatment effects to be standardized to the original DAOOH scale, such as g-computation, can improve interpretability.

For unadjusted methods, the Mann–Whitney *U*-test provides a valid approach to compare DAOOH across trial arms, especially when covariate adjustment is not preferred for the primary analysis. It is often reported without an estimand, but efforts should be made to report a corresponding probabilistic index estimate and confidence interval, while also providing a clear interpretation of that measure (e.g., the probability that a randomly selected patient in the intervention arm has a larger DAOOH than a randomly selected control patient).

Among the three regression models that enable covariate adjustment, ordinal regression provided the best fit to the SSU data as shown by residual plots and QQ-plots. This suggests ordinal regression can effectively model the underlying structure of the DAOOH data. However, the resulting odds ratio estimand is challenging to interpret. The g-computation technique estimates a causal effect by using predictions from a regression model and enables direct comparison of models which naturally would have targeted different estimands. Notably, if the assumed outcome model is not correctly specified, the resulting treatment effect estimates can be biased, but recent work has highlighted that in the randomized trial setting, this method can be quite robust and avoid bias in practice [23]. This and other recent work motivated the FDA to include g-computation as a valid method for estimating treatment effects in clinical trials, particularly for estimating marginal effects while adjusting for covariates to potentially gain power [19]. Thus, the combination of g-computation with a specified ordinal regression outcome model provides an adjusted approach which should (i) fit the skewed DAOOH outcome well, (ii) be robust to model misspecification in practice [14], and (iii) be flexible to target a specific estimand of interest which is more interpretable than the natural odds ratio that would have been targeted by ordinal regression alone. All code used for each method has been made publicly available on GitHub (https://github.com/Xinn-Wu/Statistical-Modeling-of-Days-Alive-Out-of-Hospital-An-Illustration-Using-the-SSU-Trial.git).

This study complements and extends other recent work providing guidance for trials using DAOOH outcomes. Granholm et al. [2] also discussed methodological considerations and statistical analysis methods for outcome variables such as DAOOH and Days alive without life support. Their discussion treats DAOOH as a count outcome reflecting number of calendar days out of the hospital. Their study focuses on notably different scenarios where DAOOH is concentrated at zero and maximum values, reflecting clinical populations with high rates of non-survival and full recovery. In such cases, they found that simple models can often provide good fit and interpretability. However, when exact timestamps for admission and discharge are recorded, treating DAOOH as a continuous variable yields more information during analysis. Focusing on a scenario where DAOOH values are continuously and evenly distributed across the higher value range, we found that more complex models such as ordinal regression may be needed to ensure good fit. Future studies may benefit from combining both perspectives depending on the data structure encountered.

There are limitations of this study. In some trials, an alternative version of DAOOH is used which does not set the outcome to 0 when death occurs in the time window of interest [2]. While we did not consider this version of DAOOH, it may be more appropriate for high-mortality settings like palliative care trials. We used different functions for multiple imputation in different regression models, as the rms [26] package used for ordinal regression was not compatible with the mice [25] package for multiple imputation in R. Although a limited assessment suggests that the mice function and the utilized aregImpute function for ordinal regression provide very similar results, the underlying imputation methods are somewhat different. Thus, the ordinal regression model approach may differ slightly from those of other models. In addition, we implement the multiple imputation with the default predictive mean matching method for illustrative purposes because the missing data in the SSU study is minimal. In practice, there are more sophisticated imputation methods that can be used (e.g., random forests). The g-computation technique relies on modeling the conditional mean of the outcome, making it hard to directly compare methods such as probabilistic index regression with other regression models considered in our study. Accordingly, g-computation paired with a PIM model was not illustrated. Although a brief comparison with log-transformed linear regression is provided in the Supplement, this method was not systematically evaluated. Quantile regression, which can be used to target quantile treatment effects and may be preferred when the outcome is heavily skewed, was not explored. Two-part models which accommodate boundary clustering in the outcome were also beyond the scope of this study. To further strengthen our findings, future work could include a simulation study to systematically evaluate and compare these methods, especially from the perspective of estimation accuracy and power.

## Conclusion

This study highlights that a wide range of estimands and statistical methods can be considered for trials with a DAOOH outcome. For this outcome, the Mann–Whitney *U*-test provides a simple and robust approach to report an unadjusted effect, and it can be paired with a probabilistic index and confidence interval to enhance interpretability. To report an adjusted treatment effect, ordinal regression paired with g-computation provides a strong balance of precision, model fit, and interpretability. This work provides key considerations for investigators during trial planning and analysis.

## Supporting information

10.1017/cts.2026.10786.sm001 Wu et al. supplementary material Wu et al. supplementary material

## Supplementary material

The supplementary material for this article can be found at https://doi.org/10.1017/cts.2026.10786.

## Table 1. Long description

A table with three rows and three columns. The columns are labeled Metric, Evaluation approach, and Methods to be evaluated. The rows are labeled Interpretability, Precision, and Model fit. Interpretability is evaluated on the difference scale for untransformed DAOOH using T-test, Mann-Whitney U-test, Linear Regression, Gamma Regression, Ordinal Regression, and Probabilistic Index Model. Precision is assessed using the confidence interval width for Linear Regression, Gamma Regression, and Ordinal Regression. Model fit is evaluated using diagnostic plots and RMSE for Linear Regression, Gamma Regression, and Ordinal Regression.

Navigate back to Table 1..

## Table 2. Long description

A table comparing different statistical methods and their results. The table has six rows and six columns. The columns are labeled Method, Adjustment, Estimand, Effect type, Estimate (95% CI), and RMSE. The rows are grouped into three categories: Additive model, Multiplicative model, and Probabilistic model. Each row lists a specific method, its adjustment type, the estimand used, the type of effect measured, the estimate with a 95% confidence interval, and the RMSE value. The Additive model includes T-test and Linear regression. The Multiplicative model includes Gamma regression and Ordinal regression. The Probabilistic model includes Mann-Whitney U-test and Probabilistic index model.

Navigate back to Table 2..

## Figure 2. Long description

Three scatter plots depict residuals from different regression models. Panel A: A scatter plot shows residuals from linear regression with DAOOH on the x-axis and residuals on the y-axis. The data points are scattered around the zero line on the y-axis, indicating the fit of the linear regression model. Panel B: A scatter plot displays simulated residuals from gamma regression with DAOOH rank transformed on the x-axis and residuals on the y-axis. The data points are more evenly distributed, showing a minor quantile deviation at the 0.25 quantile. Panel C: A scatter plot presents PResiduals from ordinal regression with DAOOH on the x-axis and PResiduals on the y-axis. The data points are scattered around the zero line on the y-axis, indicating the fit of the ordinal regression model.

Navigate back to Figure 2..

## Table 3. Long description

A table comparing different regression models and their estimates. The table has three rows and four columns. The columns are labeled Method, Estimand, Effect type, and Estimate (95% CI). The rows are grouped into two sections: Additive model and Multiplicative model. Under Additive model, the method is Linear regression with Standardized mean difference estimand, Marginal effect type, and an estimate of 1.37 with a 95% CI of (-0.07, 3.04). Under Multiplicative model, the methods are Gamma regression and Ordinal regression, both with Standardized mean difference estimand and Marginal effect type. Gamma regression has an estimate of 1.23 with a 95% CI of (-0.38, 2.93), and Ordinal regression has an estimate of 1.43 with a 95% CI of (0.01, 2.82).

Navigate back to Table 3..

## Figure 3. Long description

Three quantile-quantile plots compare regression models, showing alignment with a diagonal line to indicate fit. Panel A: A quantile-quantile plot for a linear regression model. The x-axis is labeled Quantiles of Normal Distribution, and the y-axis is labeled Sample Quantiles. The plot shows data points deviating from the diagonal line, indicating a poor fit. Panel B: A quantile-quantile plot for a gamma regression model. The x-axis is labeled Quantiles of Uniform Distribution, and the y-axis is labeled Theoretical Quantiles. The plot shows data points closely following the diagonal line, indicating a good fit. Panel C: A quantile-quantile plot for ordinal regression models. The x-axis is labeled Quantiles of Uniform Distribution, and the y-axis is labeled Theoretical Quantiles. The plot shows data points closely following the diagonal line, indicating a good fit.

Navigate back to Figure 3..
