# Supplementary material for: Statistical modeling of days alive out-of-hospital: An illustration using the SSU trial
Source: J Clin Transl Sci. 2026 Jul 7;10(1):e129. doi: 10.1017/cts.2026.10786 (PMC13420199; doi:10.1017/cts.2026.10786)
Supplement: Wu et al. supplementary material [file S2059866126107869sup001.docx]

**Supplementary Material for Statistical Modeling of Days Alive Out-of-Hospital: An Illustration Using the SSU Trial**

**Models and coefficient transformation**

We define $E\left[ Y \right]$ as the expected value of DAOOH, $X_{SSU}\in\{0,1\}$ as the treatment allocation, and $\boldsymbol{X}=\left( X_{1},\ldots X_{p} \right)^{T}$as observed covariates.

**T-test**

T-test can be equivalently expressed as a coefficient from a linear regression model with a single binary predictor:

$$E\left[ Y \right]=\beta_{0}+\beta_{SSU}\times X_{SSU}.$$

The estimated $\beta_{SSU}$is the coefficient of interest.

**Linear Regression**

Linear regression model includes other covariates as $\boldsymbol{X}$,

$$E\left[ Y \right]=\beta_{0}+\beta_{SSU}\times X_{SSU}+\boldsymbol{\beta}^{'}\times\boldsymbol{X}.$$

The estimated $\beta_{SSU}$is the coefficient of interest.

**Gamma regression**

Gamma regression with a log link models the log of the expected value of DAOOH:

$$\log\left( E\left[ Y \right] \right)=\beta_{0}+\beta_{SSU}\times X_{SSU}+\boldsymbol{\beta}^{'}\times\boldsymbol{X}.$$

To ensure model feasibility, the response variable should be transformed into $DOHD = 30 - DAOOH,$ so that the cases with $DAOOH= 30$ will not lead to undefined log values:

$$\log\left( E\left[ 30-Y \right] \right)=\beta_{0}^{*}+\beta_{SSU}^{*}\times X_{SSU}+{\boldsymbol{\beta}^{'}}^{*}\times\boldsymbol{X}.$$

An exponential function is used to transform the prediction back to the original scale. The estimated $exp\left( \beta_{SSU}^{*} \right)$ is the coefficient of interest.

**Comparison of gamma regression and linear regression with log-transformation**

An alternative approach that was briefly discussed in the main text is linear regression with log-transformation. It also provides a way to model DOHD:

$$E[\log(30-Y)]=\beta_{0}^{*}+\beta_{SSU}^{*}\times X_{SSU}+{\boldsymbol{\beta}^{'}}^{*}\times\boldsymbol{X}.$$

After exponentiation, $exp\left( \beta_{SSU}^{*} \right)$ represents a ratio on the geometric mean scale of DOHD. The log-transformed linear model does not directly target the mean of DOHD after coefficient transformation, because $E[\log(DOHD)]$ is generally not equivalent to $log(E[\mathrm{DOHD}]).$ Therefore, the treatment coefficient is interpreted as a contrast on the log scale, or after exponentiation, as a ratio on a geometric-mean/median.

We implemented this model as an additional comparison. The estimated treatment coefficient was similar to that from the gamma regression model (-0.27 versus -0.23), although the two models target different quantities: gamma regression with a log link models $log(E[\mathrm{DOHD}])$, whereas linear regression of a log-transformed outcome models $E[\log(DOHD)]$. For brevity, we did not include this approach in our primary analysis.

**Ordinal regression**

With a simplifying assumption of proportional odds, the regression model can be expressed as:

$$logit\left( P\left( Y\leq m | X=x \right) \right)=log\left( \frac{P\left( Y\leq m | X=x \right)}{P\left( Y>m | X=x \right)} \right)=\beta_{0m}-\beta_{SSU}\times X_{SSU}-\boldsymbol{\beta}^{'}\times\boldsymbol{X},$$

where $\beta_{SSU}$ and $\boldsymbol{\beta}^{'}$ are the same across all categories. The estimated $exp\left( \beta_{SSU} \right)$ is the coefficient of interest.

**Proportional odds (PO) assumption assessment**

The proportional odds assumption in the ordinal regression model for continuous outcomes can be assessed through the ordParallel function in the rms package. This function assesses the proportional odds assumption graphically by refitting the ordinal model as a series of binary models across different outcome cutoffs. The flatter and more stable the coefficient curves are across outcome cutoffs, the more plausible the proportional odds assumption is.

From Figure S1, we can observe that most curves are stable across the cutoffs, except for the slight curve for the troponin-I low category. Since our primary interest lies in the coefficient of the treatment-group indicator (Indicator for $X_{SSU}$), the figure does not suggest a meaningful violation of the proportional odds assumption for the treatment effect in our ordinal regression model.


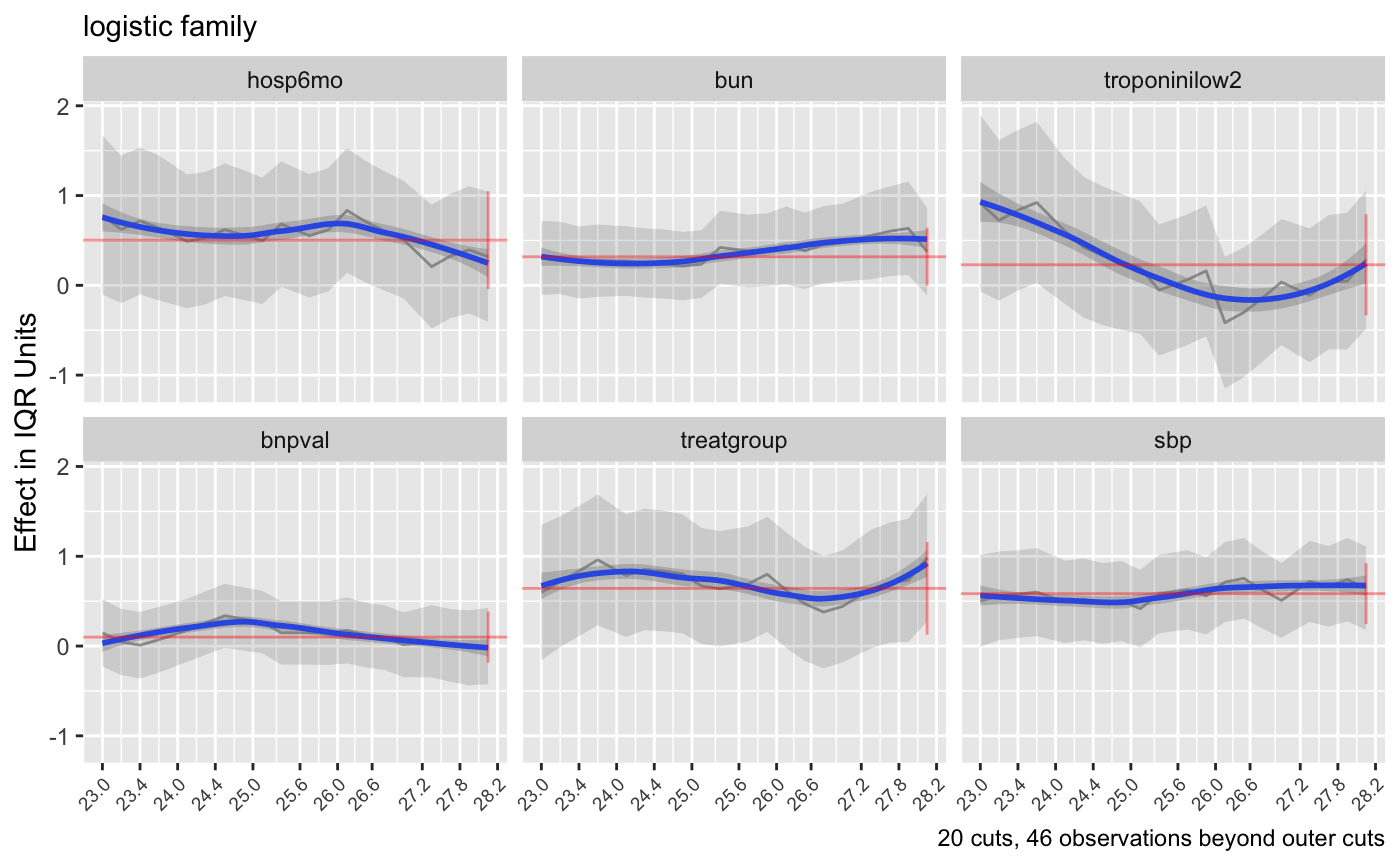


**Figure S1.** Proportional odds assumption assessment. The flatter and more stable the coefficient curves are across outcome cutoffs, the more plausible the proportional odds assumption is.

**Mann-Whitney U-test**

The Mann‐Whitney parameter can be expressed as:

$$P\left( Y_{SSU}>Y_{\mathrm{Hospitalization}} \right)+\frac{1}{2}\times P\left( Y_{SSU}=Y_{\mathrm{Hospitalization}} \right),$$

where $Y_{SSU}$ and $Y_{Hopstitalizaiton}$ are two independent observations of DAOOH from the two groups.

**Probabilistic Index Model**

The probabilistic index model for pairwise comparisons between SSU and hospitalization can be stated as:

$$logit\left( P\left( Y_{\mathrm{Hospitalization}}\leq Y_{\mathrm{SSU}} \right) \right)={\beta_{SSU}\times\left( X_{SSU}^{'}-X_{SSU} \right)+\left( \boldsymbol{X}^{'}-\boldsymbol{X} \right)}^{T}\boldsymbol{\beta}$$

where $Y_{SSU}$ and $Y_{Hospitalization}$ are two independent observations of DAOOH from the two groups. $expit\left( \beta_{SSU} \right)=\frac{exp\left( \beta_{SSU} \right)}{1+exp\left( \beta_{SSU} \right)}$ is the coefficient of interest.

**G-computation**

G-computation allows for formal marginalization over adjustment covariates to target a marginal treatment effect. It estimates the marginal causal effect by fitting an outcome model and making predictions for each observation under each treatment condition [20].

To implement g-computation, we first fit a model for a regression of outcome $Y$ on $X_{SSU}$ and $\boldsymbol{X}: E[Y|X_{SSU},\boldsymbol{X} ]$. Then we can predict the counterfactual DAOOH for each subject under each possible intervention, $Y_{S}=\{0, 1\}$ for binary treatment $X_{SSU}$. Specifically, we plug in $X_{SSU}=1$ and then $X_{SSU} = 0$ into the regression model $E[Y|X_{SSU},\boldsymbol{X} ]$ to obtain the predicted outcome. When the model is fitted on a transformed scale or uses a non-identity link, the predicted values are then transformed back to the original DAOOH scale using the corresponding inverse transformation or prediction scale. This can be completed by specifying the type argument in the predict function. (R code at GitHub link: https://github.com/Xinn-Wu/Statistical-Modeling-of-Days-Alive-Out-of-Hospital-An-Illustration-Using-the-SSU-Trial.git)

To combine g-computation with bootstrap and multiple imputation, we first generate a bootstrap sample. Within each bootstrap sample, we conduct multiple imputation and pool the model estimates. With the pooled model, we implement g-computation and calculate the standardized mean difference on the DAOOH scale. By repeating this procedure by 500 times, we can obtain the mean and quantile-based confidence interval for the G-computation results.

For reproducibility, we specified a random seed of 123 at the start of the bootstrap-MI-g-computation procedure. We did not reset the seed separately within each call to the mice algorithm, so that imputations could vary naturally across bootstrap resamples while remaining reproducible.
